# Monoamine Oxidase A and B Gene Polymorphisms and Negative and Positive Symptoms in Schizophrenia

**DOI:** 10.5402/2012/852949

**Published:** 2012-04-19

**Authors:** Beatriz Camarena, Ana Fresán, Alejandro Aguilar, Raúl Escamilla, Ricardo Saracco, Jorge Palacios, Alfonso Tovilla, Humberto Nicolini

**Affiliations:** ^1^Posgrado de Ciencias Genómicas, Universidad Autónoma de la Ciudad de México, 03100 México, DF, Mexico; ^2^Departmento de Genética Psiquiátrica, Instituto Nacional de Psiquiatría Ramón de la Fuente Muñiz, 14370 México, DF, Mexico; ^3^Subdirección Investigaciones Clínicas, Instituto Nacional de Psiquiatría Ramón de la Fuente Muñiz, 14370 México, DF, Mexico; ^4^Clínica de Genética Psiquiátrica, Instituto Nacional de Psiquiatría Ramón de la Fuente Muñiz, 14370 México, DF, Mexico; ^5^División Académica Multidisciplinaria de Comalcalco, Universidad Juárez Autónoma de Tabasco, 86040 Comalcalco, Tabasco, Mexico; ^6^Servicios de Atención Psiquiátrica, Secretaría de Salud y Grupo Médico Carracci, Carracci 107, 03740 México, DF, Mexico

## Abstract

Given that schizophrenia is a heterogeneous disorder, the analysis of clinical characteristics could help to identify homogeneous phenotypes that may be of relevance in genetic studies. Linkage and association studies have suggested that a locus predisposing to schizophrenia may reside within Xp11. We analyzed uVNTR and rs1137070, polymorphisms from *MAOA* and rs1799836 of *MAOB* genes to perform single SNP case-control association study in a sample of 344 schizophrenia patients and 124 control subjects. Single polymorphism analysis of uVNTR, rs1137070 and rs1799836 SNPs did not show statistical differences between cases and controls. Multivariate ANOVA analysis of clinical characteristics showed statistical differences between *MAOB*/rs1799836 and affective flattening scores (*F* = 4.852, *P* = 0.009), and significant association between *MAOA*/uVNTR and affective flattening in female schizophrenia patients (*F* = 4.236, *P* = 0.016) after Bonferroni's correction. Our preliminary findings could suggest that severity of affective flattening may be associated by modifier variants of *MAOA* and *MAOB* genes in female Mexican patients with schizophrenia. However, further large-scale studies using quantitative symptom-based phenotypes and several candidate variants should be analyzed to obtain a final conclusion.

## 1. Introduction

Schizophrenia is a severe and chronic mental illness with a complex clinical presentation suggesting an etiologic and genetic heterogeneity that could benefit from the definition of particular phenotypes using quantitative heritable components [1].

A recent dopamine hypothesis of schizophrenia suggests a final common pathway of presynaptic striatal hyperdopaminergia caused by an interaction between multiple environmental and genetic risk factors affecting brain function that underlie negative and cognitive symptoms [2]. Negative symptoms such as affective flattening, apathy, and poverty of speech are conceptualized as deficits in normal behaviour. Positive symptoms are characterized by hallucinations, delusions, and severe thought disorganization.

Family, twin, and adoption studies have provided evidence for genetic influences on pronounced negative symptoms in patients with a family history of schizophrenia, as well as for an association with a particular familial form of the illness [3–5].

Linkage and association studies have suggested that a locus predisposing to schizophrenia may reside within Xp11 [6–8]. However, a meta-analysis study did not confirm association between Xp11 locus and schizophrenia [9]. Based on chromosomal position and participation in catabolism of dopamine pathway, monoamine oxidase (MAO) genes have been proposed as candidate genes in the pathogenesis of schizophrenia. *MAOA* and *MAOB* genes are oriented tail to tail on the Xp11.23 chromosomal region; both genes comprise 15 homologous exons [10]. A functional polymorphism located on the promoter region of the *MAOA* gene (uVNTR) has been described and characterized by a variable-number tandem repeat (VNTR) of 30 bp sequence that expressing five alleles of 2, 3, 3.5, 4, and 5 copies [11]. Previous association analysis of this polymorphism has been studied with aggressive behaviour in schizophrenia patients [12, 13]. Also, a nonfunctional polymorphism located on exon 14 of the *MAOA* gene has been reported in relation to enzyme activity levels. It is characterized by a C for T substitution in position 1460 (C1460T) that results in an *EcoRV* restriction length polymorphism site (rs1137070) [14]. A meta-analysis study provided no evidence for an association between *MAOA* gene polymorphisms and schizophrenia [15]. However, it was reported a high frequency of a particular *MAOA* haplotype in males with schizophrenia compared with a control group [16]. Moreover, a recently study did not show association between T941G and uVNTR polymorphisms of *MAOA* and schizophrenia [17].

On other hand, low platelet MAO-B activity has been detected in schizophrenia patients [7, 18]. Interestingly, it has been published that selegiline, a selective monoamine oxidase B inhibitor in combination with antipsychotics could be helpful for treating negative symptoms in schizophrenia [19].

*MAOB* gene contains an A644G polymorphism (rs1799836) located on intron 13 that has been associated with enzyme activity in human brain [20]. On the other hand, haplotype analysis of uVNTR of *MAOA* and A644G polymorphism of *MAOB* has shown that individuals carrying the 3G haplotype have significantly lower MAOA activity levels in their brains [20].

Several studies suggest that *MAOB* gene may be implicated in the susceptibility to schizophrenia [6, 8, 21]. Moreover, a recent study found an association between a *MAOB* microsatellite polymorphism and particular clinical features [22].

The role of MAO genes in the susceptibility to schizophrenia requires further research; therefore, we analyzed *MAOA* and *MAOB* gene polymorphisms to perform single SNP association study in Mexican patients with schizophrenia.

## 2. Material and Methods

### 2.1. Sample

The total sample comprised 344 schizophrenia patients (135 females and 209 males), 267 patients from the Instituto Nacional de Psiquiatría Ramón de la Fuente (INPRF), and a sample of 77 patients from Grupo Medico Carracci selected from a study of first psychotic episode. All patients fulfilled DSM-IVR diagnostic criteria for schizophrenia and were based on a structured interview DIGS (Diagnostic Interview for Genetics Studies) Spanish version. Exclusion criteria included concomitant medical or neurological illness, current substance abuse, history of substance dependence, or history of bipolar disorder. Symptom severity was assessed before pharmacological treatment with the Scale of the Assessment of Negative Symptoms (SANS) [23] and the Scale of the Assessment of Positive Symptoms (SAPS) [24]. All relevant diagnostic information for each subject was reviewed, blind to marker genotypes, independently by H. Nicolini, A. Fresán, R. Escamilla and R. Saracco.

The control group comprised 124 healthy Mexican subjects (64 females and 60 males), without current or past psychiatric history screened-out by the DIS-Spanish version.

All subjects were from a family background of three generations born in Mexico in order to have a more homogenous sample, because there is known genetic heterogeneity in Latin American and Mexican populations [25]. The study received approval by the appropriate Institutional Review Boards of the INPRF and subjects provided written informed consent.

### 2.2. Genotyping Analysis of MAOA and MAOB Polymorphisms

Genomic DNA of the subjects was extracted by a standard procedure. The *MAOA*/uVNTR analysis was performed using the primers and conditions described by Sabol et al. [11]. *MAOA* genotyping was performed in 3% MetaPhor gels and visualized under UV light after staining with ethidium bromide. Analysis of the rs1137070, of the *MAOA* gene was performed using the primers and conditions reported by Hotamisligil and Breakefield [14]. PCR products were resolved on 3% agarose gels and visualized under UV illumination after ethidium bromide staining. The rs1799836 of *MAOB* gene was detected using the method described by Matsumoto et al. [26].

### 2.3. Statistical Analysis

Analysis was performed with the Statistical Package for the Social Science (SPSS) for Windows, version 18.0 (SPSS Inc., Chicago). A Multivariate General Linear Model (Multivariate Analysis of the Variance, MANOVA) was performed to identify differences between the quantitative variables of interest (SANS and SAPS subs-scales) between genotypes. Bonferroni's correction for multiple testing was applied (3 MAO gene polymorphisms corrected at *P* ≤ 0.016).

## 3. Results

The mean age of the 344 schizophrenia subjects was 32.6 ± 9.8 years (mean ± s.d.). The age for the onset of illness was 22.1 ± 7.1 years. The mean of SANS total scores was 10.1 ± 5.1 and for SAPS total scores was 5.6 ± 4.4.

We analyzed uVNTR and rs1137070 polymorphisms on the *MAOA* gene and rs1799836 located on the *MAOB* gene. The distribution of *MAOA* and *MAOB* genotypes for schizophrenia and control groups were in Hardy-Weinberg equilibrium (*P* > 0.05).

Allele frequencies of *MAOA* and *MAOB* polymorphisms are shown in Table 1. Two alleles containing 3 and 4 repeats (allele 1 and 3, respectively). for the uVNTR polymorphism were observed in Mexican population (Table 1). Single polymorphism analysis by gender of uVNTR, rs1137070 and rs1799836 SNPs did not show statistical differences between cases and controls (Table 1).

The presence of association of each polymorphism with SANS and SAPS subscale scores were analyzed using Multivariate ANOVA. The comparison between SAPS subscale scores and *MAOA* and *MAOB* gene variants did not show statistical differences (data not shown). However, analysis in female patients of SANS subscale scores showed an effect of genotype on affective flattening score of SANS sub-scale for rs1799836 SNP of *MAOB *gene (*F* = 4.852, *P* = 0.009). Also, a statistical significance was observed between uVNTR polymorphisms of MAOA gene and high scores of affective flattening (*F* = 4.236, *P* = 0.016, Table 2). Therefore, we investigated the existence of a potential dose response of allele 3 of uVNTR and allele G of rs1799836 on the affective flattening scores in female group. MAOA/uVNTR genotype was converted into a quantitative variable that express the number of allele 3 (0 = no 3 allele, 1 = 1 allele 3 and 2 = 2 alleles 3, and MAOB/rs1799836 genotypes was converted in based to number of G alleles (0 = no G allele, 1 = 1 allele G, and 2 = 2 alleles G). Regression analysis was significant only for uVNTR polymorphism (*F* = 8.005, *R* ^2^ = 0.056, *P* = 0.005).

## 4. Discussion

Since schizophrenia is a complex disorder characterized by a myriad of symptoms, the use of positive and negative symptom scores as quantitative traits may increase the power to detect association with candidate genes that could be important to define a molecular classification of schizophrenia subtypes.

Genetic studies have reported association between polymorphic variants of DRD2, DRD4, BDNF, MTHFR, and COMT genes and negative symptoms [27–30]. Moreover, significant relationship have been reported between DISC1 gene and the severity of delusions [31, 32], and positive symptoms and allele variants of DRD4 and SLC6A4 [33, 34].

Linkage studies have suggested a probable locus predisposing to schizophrenia within Xp11 [6, 7]. Interestingly, there are two candidate genes within this particular chromosomal region, *MAOA* and *MAOB*.

Our* MAOA* single polymorphism analysis did not show significant association with schizophrenia in agreement with previous studies [17, 35–37]. However, the present study found a trend for significance between the number of 3 alleles of uVNTR of MAOA gene and the severity of affective flattening demonstrated that an effect of dose could be related with *MAOA* gene variants. Our findings are consistent with previous studies showing an effect dose of gene variants of enzymes that participate in the metabolism of neurotransmitters associated with the negative symptoms in schizophrenia [28].

A differential metabolism of MAO-A and MAO-B enzymes in dopamine metabolism has been reported in several human brain regions. Analysis of platelet MAO activity reported association with negative symptoms in male schizophrenic patients [38]. Also, it has been suggested that an increase in the MAO-B/MAO-A ratio in the brain of schizophrenic patients could be associated with negative symptoms in structural abnormalities related to these particular clinical symptoms [39]. We observed that *MAOB*/rs1799836 polymorphism was associated with the severity of affective flattening. In particular, analysis by gender showed a high severity of affective flattening in female carriers of GG genotype. However, we did not find an effect of dose related with the number of G alleles suggesting that GG genotype could be related to this clinical phenotype. The rs1799836 has been associated with the level of enzyme activity [40, 41] and revealed gender differences in Parkinson's disease [42]. Therefore, it could be possible that genetic variation of *MAOB* suggests a gender subtype of schizophrenia.

Our findings showed that MAO gene variants may be involved in pathways contributing to a schizophrenia symptom severity. Interestingly, affective flattening and alogia are independent of medication status and persist for many years [43, 44]. Therefore, our results provide support for the hypothesis that polymorphic regions influence clinical features once the disease is present.

It was reported a haplotype analysis of uVNTR of *MAOA* and A644G polymorphism of *MAOB* showing that individuals carrying the 3G haplotype have significantly lower MAOA activity levels in their brains [45]. We observed weak LD between the regions analyzed, suggesting that haplotypes had been broken up by recombination. Interestingly, *MAOA* and *MAOB* have been reported as candidate loci subject to a recent positive selection [21].

Limitations of the present study were the small sample size, the number of MAO genetic variants analyzed, and the clinical heterogeneity of the schizophrenia patients.

In conclusion, our preliminary findings could indicate that the severity of negative symptoms might be associated by modifier variants of *MAOA* and *MAOB* genes in Mexican patients with schizophrenia, in particular, with affective flattening dimension. The complexity of psychiatric disorders requires a multidisciplinary integration of genetics, neuroscience, psychiatry, and molecular biology to obtain a final conclusion. For the moment, to gain further insight into this hypothesis, we suggest that further large-scale studies using quantitative symptom-based phenotypes and several candidate variants should be undertaken to understand the role of *MAO* genes in schizophrenia pathogenesis.

## Figures and Tables

**Table 1:** Allele frequencies of *MAOA* and *MAOB* polymorphisms in schizophrenia and control subjects.

	Schizophrenia		Controls	
	*n* = 344		*n* = 124	
uVNTR	1	3	1	3
Males	76 (0.36)	133 (0.64)	19 (0.35)	36 (0.65)
Females	112 (0.41)	158 (0.59)	44 (0.40)	66 (0.60)

rs1137070	C	T	C	T
Males	121 (0.58)	88 (0.42)	39 (0.65)	21 (0.35)
Females	143 (0.53)	127 (0.47)	84 (0.66)	44 (0.34)

rs1799836	A	G	A	G
Males	136 (0.65)	73 (0.35)	31 (0.55)	25 (0.45)
Females	185 (0.69)	85 (0.31)	77 (0.58)	55 (0.42)

Schizophrenia: Males = 209.

Females = 135.

Controls: Males = 60.

Females = 64.

**Table 2:** Effect of *MAOA* and *MAOB* genotypes on SANS subscale scores in 135 females with schizophrenia using multivariate ANOVA analysis.

SANS sub-scales	Female genotypes			*F*	*P*
*MAOA*/uVNTR	11	13	33		
Affective flattening	1.75 ± 1.1*	1.96 ± 1.2	2.47 ± 1.1	4.24	**0.016**
Alogia	1.39 ± 1.5	1.58 ± 1.2	2.06 ± 1.2	3.08	0.049
Avolition	2.04 ± 1.3	2.16 ± 1.2	2.35 ± 1.3	0.64	0.528
Anhedonia	2.07 ± 1.3	2.07 ± 1.2	2.63 ± 1.3	3.08	0.049
Attention impairment	1.54 ± 1.3	1.56 ± 1.2	1.96 ± 1.3	1.60	0.206

SANS total	8.79 ± 5.4	9.33 ± 5.2	11.47 ± 5.1	3.26	0.042

*MAOB*/rs1799836	AA	AG	GG		
Affective flattening	2.13 ± 1.1	1.85 ± 1.1	2.87 ± 1.4	4.85	**0.009**
Alogia	1.75 ± 1.3	1.55 ± 1.2	2.19 ± 1.2	1.55	0.217
Avolition	2.25 ± 1.2	1.96 ± 1.2	2.81 ± 1.4	2.98	0.054
Anhedonia	2.27 ± 1.3	2.11 ± 1.3	2.88 ± 1.3	2.20	0.115
Attention impairment	1.60 ± 1.3	1.75 ± 1.4	2.00 ± 1.1	0.68	0.506

SANS total	10.0 ± 5.2	9.23 ± 5.2	12.75 ± 5.5	2.79	0.065

*Data are presented as Mean ± SD.

Bonferroni's correction adjusted to *P* ≤ 0.016.
